# Supplementary material for: Patterns of Intron Gain and Loss in Fungi
Source: PLoS Biol. 2004 Nov 30;2(12):e422. doi: 10.1371/journal.pbio.0020422 (PMC532390; doi:10.1371/journal.pbio.0020422)
Supplement: Table S1 — Also available at http://genes.mit.edu/NielsenEtAl/. (4.3 MB ZIP). [file pbio.0020422.st001.zip › NielsenEtAl/html/685.html]

AN3578.1.NCU07418.1.MG04405.1.FG06839.1


```
 CLUSTAL W (1.82) Multiple Sequence Alignments - Introns Inserted


Sequence 1: NCU07418.1	747 aa
Sequence 2: MG04405.1	740 aa
Sequence 3: FG06839.1	788 aa
Sequence 4: AN3578.1	734 aa
Alignment Length: 843 aa
Number Identitical Residues: 302 aa
Alignment Score (without introns) 15689


MG04405.1 	MVSDETYEICLPILEDESLDDEDKTDKLEELLRKETSLTGNSLENAVLDALWRYRDGGNI
NCU07418.1	MVSDETYDICLPVLEDPDLDDEEKTDKLQDLLRERTTLSGQALDNAVLDAMWRFRDGGGT
FG06839.1 	MVSDEHFEMCLPVLQDATLEDEDKTDRLEELFRKETNLTGTSLDNAILDALWRYRDSGGS
AN3578.1  	MVTEELLEDCLQILQDKSLDEEDQVEKIEEFLREKTSLSGTSLENAVLDILWRQRNRTLP
          	**::*  : ** :*:*  *::*::.:::::::*:.*.*:* :*:**:** :** *:    

MG04405.1 	AASPPPIRQTILRRPSPASWR-ANSGTPLSGSPRLGVSPLAPPGFVPTP--FGRNVVSST
NCU07418.1	ATSPPSIRQSILRRPSPAPWR-GSS-TPLSSSPRLSVSPLAPPGYVPTN--FGR-AMSST
FG06839.1 	SASPPPIRPAILRRPSPASWR-GSP-TPMSGSPRLAVSPLAPPGFVPST--FGR-TISST
AN3578.1  	DSSPPPPRHTVIRRSSPAPWQMARSSTPLSPHSNLGTSPGSSSWLQSSKGGISRPPLSST
          	 :***. * :::**.***.*: . ..**:*  ..*..** :..   .: ..:.*  :***

MG04405.1 	ASPFGSPRPSPRLAFAAPIIPNSPNLNAYEFAT--DAPSQETFGDMQND-NVDWLTN-DD
NCU07418.1	ASPFGSPRPSPRLAFATP-VPHSPNLNAYQFAANDEPPSQEVFGDYQSD-NVDWLVSDDD
FG06839.1 	VSPFSSPRASPRMALATPVIPHSPNLNAYEFAS-DPTPATEILGEYQTE-NVEWLVS-DD
AN3578.1  	VSPFTSPRPSPRLALAQP-IPHSPNLNAYEFSD-QQSHVSDFYGDLGSDSNVDWLVA-DD
          	.*** ***.***:*:* * :*:*******:*:  . .   :  *:  .::**:**.  **

MG04405.1 	AVSVTSSIG---TGSALNAAAPEYAPS--QQVDMTPYDMLRSILGPSRSDEEIDAALAMH
NCU07418.1	RLSVTSSIG---GSSGLNVAAPEFVST--QQADMSPFDMLRSILGTSKTDDEIGAALAMH
FG06839.1 	AASVTSSVS---TPSALNAAAPEFSSMTAQSADMSPYDMLRSILGQTKTDDEIEAALAQN
AN3578.1  	AMSTTSSVGGLSMHGGLSATAPEFVPD------MSPHDILRTVLGDKRSNEEIESALEAN
          	  *.***:.. :  ..*..:***: .       *:*.*:**::** .::::** :**  :

MG04405.1 	GYDLGAIITAFMDTVDSSAMQVAQTEEPKTNGLLIGKSMTPDA-RPSTPADQ-RSGIICK
NCU07418.1	GYDLSATVASIMDDPAQDDVSLSGQND-EARVVVIGKSMTPDQ-RPATPADQQRSGVICK
FG06839.1 	GYDLSATIVSFMESQTGDNQPGGVSLE-EPKNVLIGKSVD----RPSTPLGQQKSGVICK
AN3578.1  	GYDLGATIAALTQGADAGAAPSLPDDS----RVVVGKSMTMEPPKSTSTPGHNRSPVVCK
          	****.* :.:: :    .        .     :::***:  . .:.::. .:.:* ::**

MG04405.1 	FFLAQGQCLRADCRFSHDLSNHVCK2YWVAGNCLAGTTCIFSHDPVHLLNKLSVDGTNTP
NCU07418.1	FYLSTGQCLRSDCRFSHDLSNHICK2YWVAGNCLARDTCVFSHDPAHLVNRLHIDGSGTP
FG06839.1 	FYMSTGQCLRADCRFSHDLSNHLCK2YWVMGNCLAGETCIFSHDPAKLMNKLALDGASTP
AN3578.1  	YWLSTGQCLRADCRFSHDLTSHLCK2YWVMGNCLAGDGCPFSHDPSALIANLSVDG-NSS
          	:::: *****:********:.*:** *** *****   * *****  *: .* :** .:.

MG04405.1 	PPSHKGGISIQVQDMN-SFPSLR--PATPEHLGGRASGSY------FPSMGVTPPPGFKI
NCU07418.1	PTHYS---TVNVQDFN-SFPSLQ--PGTPEQLHVFPASAN------TPAVGITPPPGFKS
FG06839.1 	PSKQG---GLQLGDLN-SFPSLR--PGTPDHLSGFAGSNS------FPGVGITPPPGLKH
AN3578.1  	ATSAG--IAFQVDNAPDAFPPLQSTPGSSEQWAGQLGSKYPGYLYGGPGGKNAPHLGGKR
          	..       .:: :  .:**.*:::*.:.::     ..  ..   . *.   :*  * * 

MG04405.1 	PGLAGENLRPRSRPSSRQQQRKEPVQTAPSLDDAEAFPSLGAASAKATSKKQHGKRSSQG
NCU07418.1	HHNYGYPDRPRSRPGSRHQQ-KEVPQTVPSPDDADAFPTLGAAIAKQ-SKKHHGKRGGHN
FG06839.1 	FQGTASP-RARSRPGSRHQP-REPDISAPALDDNDAFPSLGSASMKQ-GKKHHGKRGGHG
AN3578.1  	SGSMTNLSRPHSRPGSRHQH-RELNPTALSVDDPDAFPTLAAVNAKNSGKKNHGRKNRDN
          	        *.:***.**:*  :*   :. : ** :***:*.:.  * :.**:**::. ..

MG04405.1 	HHKEGLISS---------SLADIVKMSPSPTPEAKLVSRKMGRTVSSTSIRNGENSAAAQ
NCU07418.1	HTGSGSSSSKEIASPGPSTLADIVKMSPSPVNINLRPNGKLNRNGSSTSIRNGENSAAAQ
FG06839.1 	HGHKENMAPN--------SLADIVKMSPSPTPVRP-ELKKMGRNGSSTVARNGENSPAAQ
AN3578.1  	NTTRDNMPT---------SLADVVRMSPAPASGGK---GKPSSKNNQTKGR--ENSAAAQ
          	:      ..         :***:*:***:*.        * . . ..*  *  ***.***

MG04405.1 	SIPSPKHIPWLETGEKANKAYLKARQDAIKHGGLRNKFLQS2YVSAAQAWNRNDARAAKA
NCU07418.1	AIPPPKHIPWLETGEKANKAYLKARQEAIKHGGLRNKFLQS2---AAQAWNRNDARAAKA
FG06839.1 	AIPSPKHVPWLETGERANKAYLKARQEALKHGGLRNKFLQS2---AAQAWNRNDARAAKA
AN3578.1  	SIPAPQHIPWLETGSRTNQQYIKYRTEAIRHGTVRNKFLQS2---AAQAWNRNDARAAKA
          	:**.*:*:******.::*: *:* * :*::** :*******    ***************

MG04405.1 	LSLRGQSENDLMRRAHREAARELYEERNK---NNNNNSVSSEFYVDLHGLHPEEAVEYLE
NCU07418.1	LSLRGQSENDLMRKAHREAASLLYEERNK---NNGS---CPEIYVDLHGLHPEEAVEYLA
FG06839.1 	LSLRGQSENDLMRKAHREAARELYEERNK---DNSQ---GLELYVDLHGLHPEEAVEYLE
AN3578.1  	LSLRGQAENEAMRKCHREAARQLYEERNKHLLNAGLDDASEELYVDLHGLHPEEAIEYLE
          	******:**: **:.*****  *******   : . ..   *:************:*** 

MG04405.1 	KVLLENEKE-IRPIYAITGTGHHSKNGKDKVGKAIRGFLNEWRYAFREFSVPGDQR-NMG
NCU07418.1	GILTENTSE-SRPIYAITGTGHHSKNGKDKVGKALRTFLNEWRYAYREFSVPGDRN-SMG
FG06839.1 	KILMENSRE-SQPIYAITGSGHHSKNGKDKVGRAVRNFLNEWRYAYREFSVPGDRN-NMG
AN3578.1  	KILLKHAREGRRIIYAITGTGHHSKNGKDKIGKAVKAWLNEWKYLFREFSVPGERGGYVG
          	 :* ::  *. : ******:**********:*:*:: :****:* :*******:: . :*

MG04405.1 	GILGIDARSYDKSLAR-------KSSQQEPVDILAQGKEIGEGKVKLL~VRD--TTKEP-
NCU07418.1	GILGIDARSWDKSLSQDGVSLVKKDEPKDDVDILSQGVEIGEGKVKLL~VRDPMAAKEP-
FG06839.1 	GILGIDARSWDKSLAKDG----EGEDDKQEVDILSQGVEIGDGKVRLL0CLKSLITDLMS
AN3578.1  	GILGIDPSSYDKTLAKN-------LEAEANGDEDASQPVLTMGKIQLL~KRGDIESQQ--
          	******. *:**:*::.        . :   *  :.   :  **::**       :.   

MG04405.1 	----------------~-------------------------------PKGPAGYRGR--
NCU07418.1	----------------~-------------------------------PKGPASHRGR--
FG06839.1 	TISLVLLVTNKVVANK0VLDGTGIVICLVGPLLHGIADTSNVMYLAYDPKDDGKYSFKRH
AN3578.1  	----------------~-------------------------------------------
          	                                                            

MG04405.1 	-------
NCU07418.1	-------
FG06839.1 	TSPKDLP
AN3578.1  	-------
          	
```
